# Indigenous knowledge and use of lichens by the lichenophilic communities of the Nepal Himalaya

**DOI:** 10.1186/s13002-017-0142-2

**Published:** 2017-02-21

**Authors:** Shiva Devkota, Ram Prasad Chaudhary, Silke Werth, Christoph Scheidegger

**Affiliations:** 1 Swiss Federal Research Institute WSL, Zürcherstrasse 111, CH-8903 Birmensdorf, Switzerland; 2 Central Department of Botany, Tribhuvan University, Kirtipur, Kathmandu, Nepal; 3 Research Centre for Applied Science and Technology (RECAST), Tribhuvan University, Kirtipur, Kathmandu, Nepal; 4 Institute of Plant Sciences, University of Graz, Holteigasse 6, 8010 Graz, Austria

**Keywords:** Ethnolichenology, Use values, Limbu and Sherpa ethnic groups, Ethnoveterinary

## Abstract

**Background:**

The aim of the study was to document the prevailing indigenous knowledge and various uses of lichens among the lichenophilic communities in the hills and mountainous settlements of Nepal.

**Methods:**

Ethnic uses were recorded during twelve field trips, each of roughly 15 days in three consecutive years, through direct questionnaires administered to 190 respondents. Lichen samples were identified applying microscopic observation and thin layer chromatography (TLC). Voucher specimens of identified species are deposited at TUCH (Tribhuvan University Central Herbarium) in Nepal.

**Results:**

Lichens are being used in several ways by different communities of Nepal. We recorded the ethnic use of seven species of lichens belonging to four families (Parmeliaceae, Physciaceae, Ramalinaceae and Usneaceae) and six genera (*Heterodermia, Everniastrum, Parmotrema, Ramalina, Thamnolia* and *Usnea*) among the Limbu, Sherpa, Lama, Gurung, Rai, Dalit, Tamang, Chhetri and Brahman communities. The present study revealed six use values namely; Medicinal value (MV), food value (FV), ritual and spiritual value (RSV), aesthetic and decorative value (ADV), bedding value (BV) and ethno-veterinary value (EVV) from different parts of Nepal. Three lichen species, *Everniastrum cirrhatum, E. nepalense* and *Parmotrema cetratum* were consumed by the Limbu and Rai communities. The Limbu and Sherpa ethnic groups are regarded as most lichenophilic communities while respondents from Brahman, Chhetri and Tamang communities showed less interest in lichen uses.

**Conclusions:**

The present study contributes to document traditional knowledge on various uses of lichens among nine communities with three different cultural background, inhabitants of eight different altitudinal levels of Nepal. Regarding the six values as identified from this research, significant difference (*p* = <0.05) were found along altitudinal gradients or locations of the settlements, cultural groups and ethnicity of the respondents.

**Electronic supplementary material:**

The online version of this article (doi:10.1186/s13002-017-0142-2) contains supplementary material, which is available to authorized users.

## Background

Indigenous knowledge is the local knowledge inherited by indigenous and local communities that is unique to a culture or society. The scientific exploration and documentation of indigenous knowledge on wild resources are important tools to understand traditional living and food systems of local inhabitants [1]. It is estimated that globally, around one billion people consume wild foods in their diet [2] among them wild plants play a crucial role in the subsistence strategy of rural communities in developing countries [3]. Lichens are being used in traditional foods and medicines since millenia and also play vital roles in ecosystem function and human welfare [4]. During the mid eighteenth century, regular crops were badly affected in Europe by frosts and droughts causing famine, and as a consequence, lichens were used for food because of their easy availability, cheapness and nutritive value [5]. Most of the lichens are non-poisonous although some exceptions exist. *Letharia vulpina*, *Cetraria pinastri, Bryoria fremontii* and *B. tortuosa* are well known poisonous lichens contain vulpinic acid or pinastrinic acid [6, 7].

Nepal, though a small landlocked country with an area of 147,181 sq. km possesses a diverse socio-cultural heritage with 125 ethnic groups living in five physiographic regions starting from 60 m to 8,848 m from above sea level [8]. The topographic, climatic and ecological diversity makes this area a habitat where Non-timber forest products (NTFPs) are of great importance to local ethnic groups, contributing towards food security, local economy, traditional health care practices, and cultural values [9]. Forests provide on-site ecological benefits and/or services to the local communities and each community utilizes those services in different ways and practices [10].

Several publications have documented the inventory and ethnic uses of higher plants from different regions and among various tribes of Nepal [11, 12]. In contrast, the lichen flora and traditional utilization patterns and market potentiality of lichens has hardly been explored. A total of 792 species belonging to 187 genera of lichen-forming fungi have been reported from Nepal [13] but many lichen species have yet to be discovered [14]. While documenting indigenous knowledge on uses of higher plants, respondents also mentioned lichens, but lichens were not identified to the species level [15–18]. Uses of lichens particularly among the Limbu, Rai and Sherpa ethnic groups, inhabitants of Eastern part of Nepal has been highlighted in a few studies [19–22]. Here, we document indigenous knowledge on the values of lichens for various uses in different communities from three mountainous districts of Nepal.

## Methods

### Study site

**Fig. 1:**
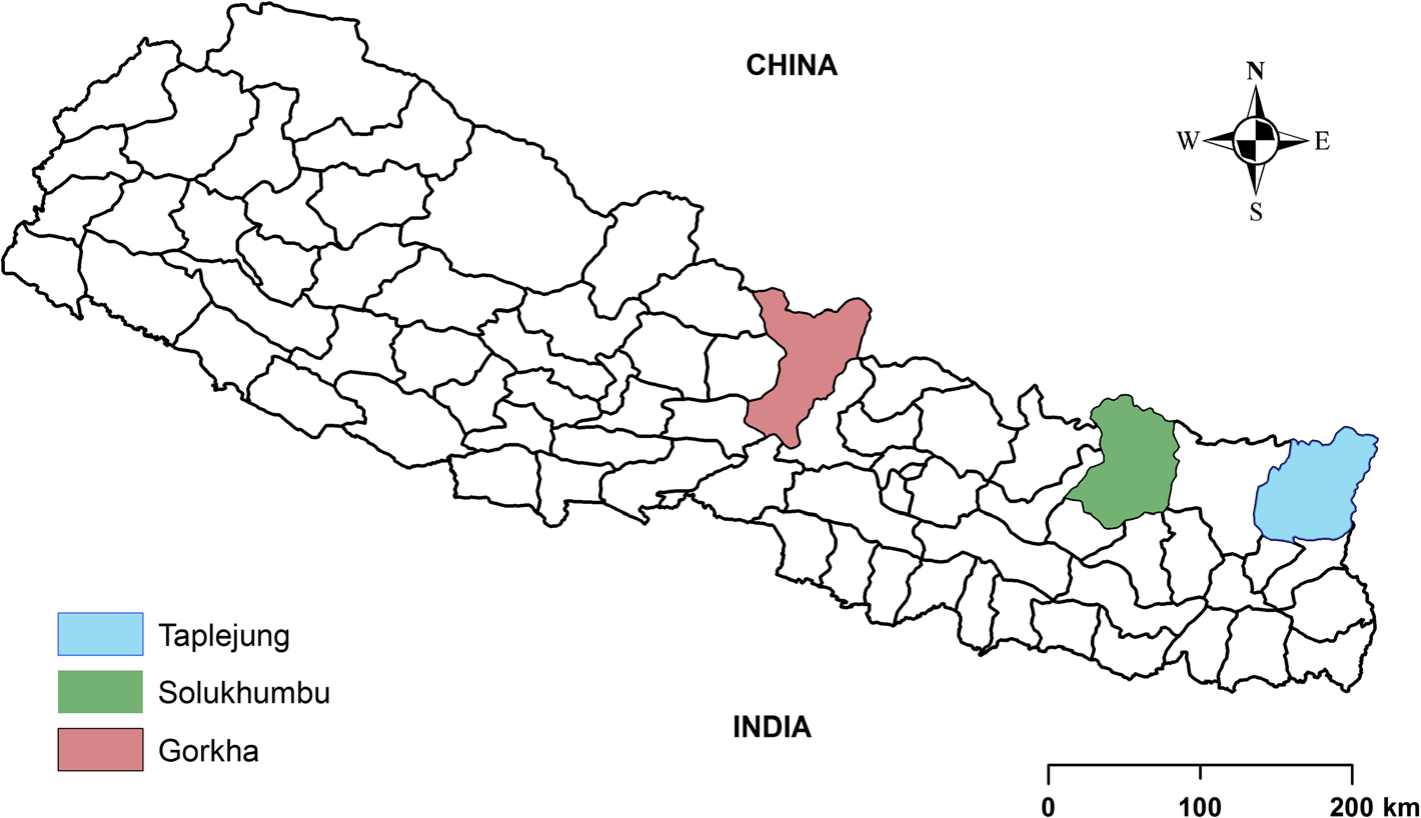
The map of Nepal showing three studied districts

The present study was conducted in three districts (Taplejung, Solukhumbu, and Gorkha) from two different regions (Eastern and Western) of Nepal (Fig. 1). The authors (SD, RPC) have accumulated sound knowledge on ethnic values of wild resources during their frequent visits to several mountainous Districts of Nepal prior to this research. Prior information played instrumental roles in selecting research areas. Eight field surveys mainly during summer season, roughly 15 days in duration, in the 22 different village development committees (VDCs) were carried out during 2011–2013 to collect ethnolichenological data. The following areas were selected to document ethnic uses of lichens: Olangchung Gola and Ghunsa Valleys located in Kangchenjunga Conservation Area of Taplejung District (N27°37′32.6958″, E87°46′34.7982″); Dudhkoshi and Dudhkunda Valleys located in Sagarmatha National Park (SNP) and surroundings of Solukhumbu District (N27°47′27.5028″, E86°39′39.9882″) in Northerneastern Nepal. Limbu, Rai, and Sherpa are main dwellers in these areas and for these communities, lichens play an important economic and cultural role. In Taplejung District, Phungling, Hangdeva, Phurumbu, Linkhim, Tapethok, Lelep, and Olangchung Gola, Village Development Committees (VDCs) were visited. In Solukhumbu, the settlements of Salleri, Beni, Taksindu, Jubing, Chaurikharka, Namche, and Khumjung VDCs were visited.

The Nubri and Tsum Valleys of Gorkha District (N28°28′35.0178″, E84°41′23.1″) located in Manaslu Conservation Area from Western part were selected as transition zone between Eastern and Western Himalayas. Scientifically, this area is poorly explored and less is known about biodiversity or social and cultural aspects from this valley because of its remoteness. In Gorkha, the VDCs of Lapu, Uiya, Sirdibas, Bihi, Prok, Samagaun, Chumchet, and Chekampar were visited. Gurung, Chhetri, and Sherpa are the main indigenous dwellers in this area.

### Field survey and data collection

Random and snowball sampling methods were employed to collect primary data [23, 24]. Respondents, who either collected, bought or at least used lichens even for a single purpose were selected for face-to-face interview using semi-structured questionnaires (Table 1). In some additional cases, though, respondents were not aware of any uses of lichens; their name, ethnic group, age, religion were asked for the further analysis to know the demographic nature of such respondents. Respondents were not forced to give their real names, answers to all the questions from a list and were not asked as in question order. Prior informed consent [25] was taken verbally from key informants including local healers before documenting their traditional knowledge on various uses of lichens. Group discussions, informal meetings while staying with local communities and field observations with key-informants were carried out. Species recommended during participatory field observations were collected. For each species they identified, informants were asked to give details of their knowledge on uses and spiritual/ritual values and these were cross-checked at least with three respondents for their validity.

**Table 1:** Questionnaire used for the collectors

1	From where do you collect *Jhyauu*?
2	During which months do you collect these *Jhyauu*?
3	Which family members are involved in collection of *Jhyauu*?
4	What type of forest you prefer to go for the collection?
5	What amount do you normally collect in a day?
6	How do you collect, dry and store?
7	What are the collecting tools?
8	What are the uses of *Jhyauu*? Medicine (veterinary/human health or others)
9	How do you prepare medicine and use them?
10	What are additional uses? Others – poisonous, edible, rituals, incense, etc. (in details)
11	How can we minimize the poisonous nature of *Jhyauu* so that they can be suitable to eat? (If poisonous and used as medicine)
12	Is the available amount of *Jhyauu* sufficient for your local demand?
13	Has the need of *Jhyauu* increased over the past couple of years?
14	Is the amount of easy accessible Jhayuu stable over the years?
15	If not, can you compensate the decline of *Jhayuu* by accessing more remote areas?
16	Does quality of *Jhyauu* matter?, which are the quality criteria other than species?
17	Are these *Jhyauu* occurring widely in your area? Specification: Rare/common/abundant
18	Have you marked any type of *Jhyauu* eaten by animals or used by animals and birds for their nest?
19	Do you know about rule and regulation of *Jhyauu*?
20	Others, if any?

Frequently seen and field-identified species were listed and unidentified species were brought to the laboratory of Central Department of Botany, Tribhuvan University, Kathmandu, Nepal for the identification. In Nubri and Tsum Valleys, the interviews with older people from the Sherpa ethnic group were carried out in *Bhotia* dialect with the help of a local translator. In Chekampar, Nile and Chuley settlements of Tsum Valley and Olangchung Gola settlement in Taplejung, the semi-structured interviews were carried out for about 15 min in the evenings when the locals had finished their daily work. Phungling, a district headquarters and also the gateway of district is the main commercial hub of Taplejung. Lichen specimens being sold in weekly markets were collected for identification.

To calculate the statistical significance of differences between uses and variables characterizing the local population (location, age group, sex, caste and ethnicity), Pearson’s Chi-squared test (*χ* ^2^) was applied [26] using the statistical software R [27].

### Lichen identification

The lichen specimens were identified by studying morphology, anatomy and lichen substances. Specimens were brought to the Swiss Federal Research Institute WSL, Switzerland where chemical substances were identified with standard thin-layer chromatography (TLC) techniques [28, 29]. Identified specimens were stored in the Tribhuvan University Central Herbarium (TUCH), Kirtipur, Kathmandu, Nepal.

## Results

### Demographic characteristics of the informants

A total of 190 informants (87 male and 103 female) representing seven age groups (<12, 13–24, 25–35, 36–47, 48–59, 60–71, >72 years), nine communities (Brahman, Chhetri, Dalit, Gurung, Lama, Limbu, Rai, Sherpa and Tamang), and three cultural backgrounds (Buddhism, Hindu, Kiraat) from eight altitudinal levels (≤1000 m, 1000–1400 m, 1400–1800 m, 1800–2200 m, 2200–2600 m, 2600–3000 m, 3000–3400 m and ≥3400 m) were approached (Additional file 1). Of the total 190 informants, 82% (*N* = 156) know at least a single use of lichens or have heard about their uses. These 156 respondents were interviewed in detail using the semi-structured questionnaires.

### Vernacular names

Highly used vernacular names for lichens are ‘*Jhyauu’* or (translation: unnecessary stuff) and ‘*Jhulo’* (translation: brittle things for the ignition) and even between the two, the most generalized name is *Jhyauu.* In addition, we documented 16 vernacular names used by indigenous peoples and ethnic groups in different parts of Nepal (Table 2). The vernacular names are derived mostly from gross morphology, life forms and palatability. Limbu and Rai called lichens as *Yangben*. Two different names ‘*Maangmaa’* and ‘*Myann’* among Sherpa ethnic group referred to edible and inedible lichens, respectively. Interestingly, it was found that people considered *Thamnolia vermicularis*, locally called *Dankini Chyau* (Witch mushrooms) as a mushroom species. Not a single respondent considered it a lichen. It was a big surprise for them to learn their real nature.

**Table 2:** Vernacular names used by indigenous people of Nepal for lichen species

Vernacular name	Meaning	Thallus organization or species	Caste or language
Jhyauu	Unnecessary stuff	All	Nepali
Jhulo	Brittle things	Foliose	Nepali
Tarey	Look like stars	Crustose	Nepali
Dankini Chyauu	Witch mushroom	*Thamnolia vermicularis*	Nepali
Maangmaa	Edible stuff	All	Sherpa, Lama
Myann	Inedible stuff	All	Sherpa
Yangben	No specific meaning	All	Limbu, Rai
Lunhokva	No specific meaning	All	Bantawa Rai^a^
Atrong Carpo^b^	Hay/Thread like	*Thamnolia vermicularis*	Himali Bhotia
Shingdrak^b^	On tree trunk	Fruticose, Foliose	Himali Bhotia
Chodrak^b^	On marshy habitat	Fruticose	Himali Bhotia
Dhodrak^b^	On rock	Crustose	Himali Bhotia
Shingbal, Ser Kue, Thangbu, Balte^b^		*Usnea longissima*	Bhotia/Kham
Budhnaa^c^		All	-

^a^Bantawa Rai is an ethnic group within Rai community [77]

^b^Additional names reported by Lama et al. [15]

^c^Name mentioned in Forest Regulations, 1995 [31]

### Collection methods

Collection was started after the end of monsoon period, i.e., August - November. People from the eastern parts of Nepal were familiar with the uses of lichens since long ago, though this use has not been documented scientifically. Personal communication with a key informant (Mr. Bharat Limbu, age 61), from Tapethok Village, Taplejung recalled his early days and mentioned that his grandfather was also collecting *Yangben* for food. The collection of lichens was found to be crucial in the lower belt of Taplejung, where most of the households collect lichens which form important parts of their diet during Dashain, Tihar and Maghey Sankrati festivals. Collectors collect in nearby forests after lunch (around 9 AM) and spend around 6–8 h for the collection. Usually at least two people often of the same family, or close neighbors work together for the collection. Children and adults, both males and females, participate in the collection. Even students collect lichens while returning home after their school as they pass through forests or pastures. In our sites, mostly they used hand and sometimes stick to pick up lichens from the substratum. Lichens are collected in a sack or plastic bag. Once returned home, they removed unwanted lichens, and litter.

### Uses of lichens

**Fig. 2:**
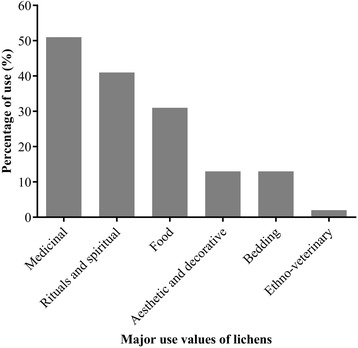
Percentage distribution of lichen uses among the respondents

Our study revealed six uses of lichens in the study areas. Medicinal value (MV), ritual and spiritual value (RSV), food value (FV), aesthetic and decorative value (ADV), bedding value (BV) and ethno-veterinary value (EVV) are the major uses of lichens. Among the 156 respondents, use percentages were high (51%) for medicinal values (MV) and low for ethno-veterinary value (2%). Ritual and spiritual values (RSV) and food value (FV) were represented with 41% and 31%, respectively. For aesthetic and decorative values (ADV) and bedding values (BV), use percentage was 13% (Fig. 2).

**Fig. 3:**
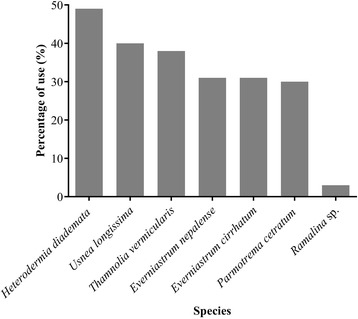
Use percentage of important lichens species among the respondents

Seven lichen species are particularly important among the respondents for various uses. Among these, use percentage was highest for *Heterodermia diademata* (49%) and lowest for *Ramalina* sp. (3%). Use percentage of *Everniastrum cirrhatum, E. nepalense*, and *Usnea longissima* were 40%, 38%, and 30%, respectively. For *Parmotrema cetratum* and *Thamnolia vermicularis*, use percentages were 31% (Fig. 3).

Respondents from nine communities used seven lichen species to meet their demands regarding the six lichen uses (Table 3). Among those, *Heterodermia diademata* and *Ramalina* species were used for medicinal values, *Everniastrum cirrhatum, E. nepalense,* and *Parmotrema cetratum* for food value, *Usnea longissima* for the ritual, spiritual, aesthetic values and as bedding materials, and *Thamnolia vermicularis* for the aesthetic and spiritual values. Interestingly, *H. diademata* was used by all ethnic communities.

**Table 3:** Lichen species used by nine ethnic communities for different use values

Species	Brahman	Chhetri	Dalit	Gurung	Lama	Limbu	Rai	Sherpa	Tamang	Use Values
*Heterodermia diademata*	+	+	+	+	+	+	+	+	+	MV
*Everniastrum cirrhatum*	+	-	+	+	-	+	+	+	+	FV
*E. nepalense*	+	-	+	+	-	+	+	+	+	FV
*Parmotrema cetratum*	+	-	+	+	-	+	+	+	+	FV
*Ramalina* sp.	+	-	+	-	-	+	+	-	-	MV
*Thamnolia vermicularis*	-	+	-	+	+	+	-	+	+	RSV
*Usnea longissima*	-	+	-	+	+	+	-	+	-	RSV, BV, ADV

*MV* medicinal value, *FV* food value, *RSV* ritual and spiritual value, *BV* bedding value, *ADV* aesthetic and decorative value

### Lichens and ethno-medicines

Forty nine percent of respondents (*N* = 77) used *Heterodermia diademata* to treat wounds and to stop bleeding after an injury. Extracts or juices of *Artemisia vulgaris* or *Eupatorium odoratum* mixed with this lichen species are used to cure fresh wounds or cuts. Regarding the medicinal value (MV), a significant difference (*p* = <0.05) was observed with altitudinal gradients, cultural groups and ethnicity (Table 4). Two respondents (>72 years) from Hangdewa and two (60 and 71 years) respondents from Phurumbu, Taplejung District used *Ramalina* sp. as antiseptic tincture to heal wounds.

**Table 4:** Significant of difference among use values and respondents with different demographic characters

Lichen uses		Altitudinal levels	Religion	Ethnicity
	df	7	2	8
Medicinal	*χ* ^2^ value	21.800	26.275	46.000
	*P*-value	<0.05	<0.05	<0.05
Ritual and spiritual	*χ* ^2^ value	87.733337	47.5	113.4
	*P*-value	<0.05	<0.05	<0.05
Food	*χ* ^2^ value	90.3333	63.378	214.875
	*P*-value	<0.05	<0.05	<0.05
Aesthetic and decorative	*χ* ^2^ value	48.7143	21.7143	69.4286
	*P*-value	<0.05	<0.05	<0.05
Bedding	*χ* ^2^ value	92.1429	12.95	35.1429
	*P*-value	<0.05	<0.05	<0.05
Ethno-veterinary	*χ* ^2^ value	21	6.0	24.0
	*P*-value	<0.05	<0.05	<0.05

### Lichens in Nepalese cuisine

**Fig. 4:**
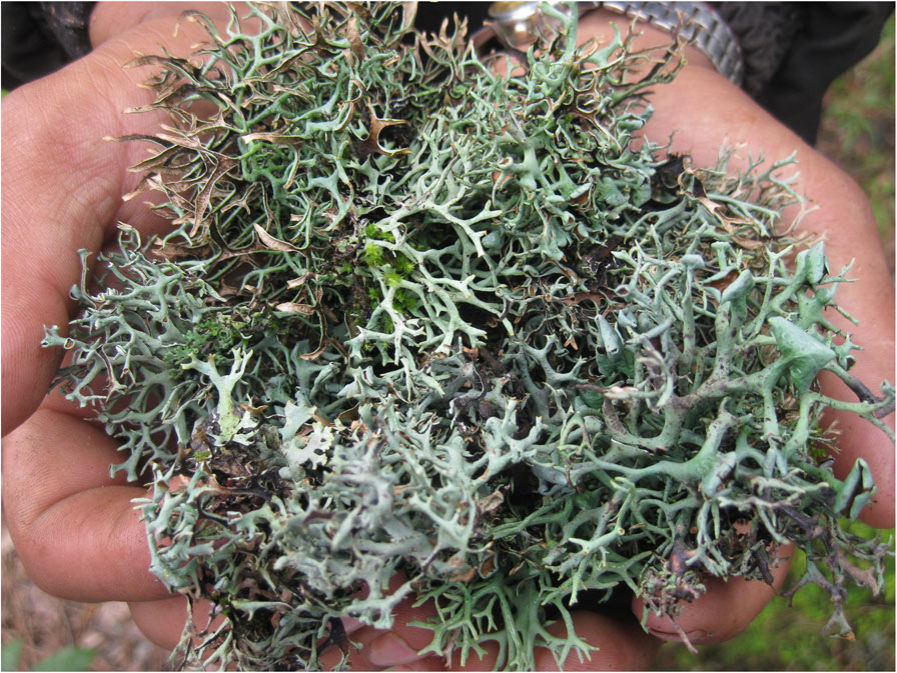
Freshly collected *Everniastrum nepalense*

Regarding the food value (FV), a significant difference (*p* = <0.05), was observed according to altitudinal levels of the studied areas, religion and ethnicity (Table 4). Although lichens occur from outer Tarai to the high mountains, the use of lichens in cuisine is observed only among Limbu, Rai and partly with Sherpa and Tamang ethnic groups. These groups are major dwellers of Eastern midhills and mountainous regions of Nepal. Major dishes prepared with steamed lichens are pickle, curry, soup and sausages. Twenty six percent of the respondents prepared dishes of *Everniastrum nepalense*, *E. cirrhatum* and *Parmotrema cetratum.* Freshly harvested (Fig. 4) or purchased lichens (about 250 g) are boiled for about 1 h with ash (about 50 g), rinsed in clean cold water until any yellow color is gone, sun dried on bamboo woven baskets and placed in closed containers in a dry place. Boiled and dried lichens can be stored for a year. For bread making, boiled, dried and powdered lichens are mixed mainly with wheat or barely flour (1:3 mixed lichens and flour).

**Fig. 5:**
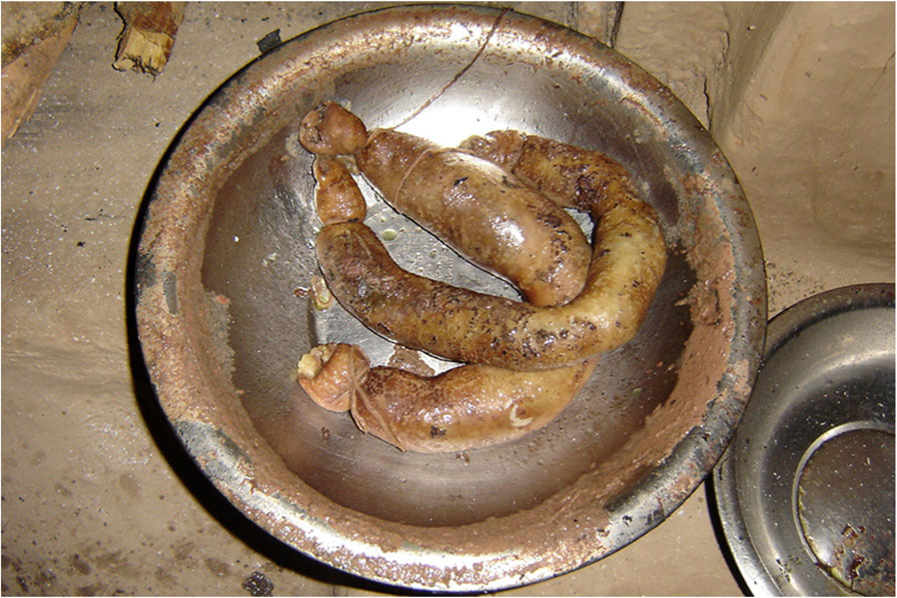
Sausage prepared with a mixture of lichens and other ingredients in pork’s big intestine

Limbu people prepare a special traditional dish called *Sargyangma* made of lichens to celebrate their special gatherings and social functions like Dashain, Tihar and in some cases, when a pig is slaughtered in the community (Fig. 5). *Sargyangma* is a kind of sausage made up of minced pork, pork’s blood, eggs, fat, rice grains (optional), spices, onion, garlic, chilly, turmeric powder, ginger, salts and lichens inserted in the pork’s big intestines. Filling of ingredients should not be compact. Boiled and dried lichens is ground fine and mixed with other ingredients. Sometimes, *Sargyangma* is specially prepared for old elderly people during the festivals who cannot eat meat because of their weaker teeth.

*Everniastrum cirrhatum*, *E. nepalense* and *Parmotrema cetratum* were much preferred species and found to be sold in local weekly markets in the district headquarter of Taplejung. Our study found that since the last 5–7 years, the demand for edible lichens is rising, especially from abroad. Thousands of Rai and Limbu families from Eastern part of Nepal are living abroad (mainly in UK and Hongkong) and they want to taste lichen during their festivals. Lichens are also considered as the best gift item in such communities.

### Ritual, spiritual and aesthetic values

*Thamnolia vermicularis* was used on the belief that this lichen wards off evil spirit and maintains peace at home and among family members. Respondents kept a handful of lichens above the main entrance of a house. Twenty two respondents (male 10, female 13) from six villages (Hangdewa 1, Lelep 1, Linkhim 2, Phurumbu 3, Tapethok 4 and Olangchung Gola 12) of Taplejung District used this lichen. Such practice was common among the Buddhist and Kirant cultural groups. Similarly, people of Olangchung Gola and Ghunsa used *Usnea longissima* to clean religious cups like butter lamps (*Diyo* in Nepali) and water bowls made of silver. With wet *U. longissima*, pots are scrubbed to remove stains.

*Usnea longissima* and *Thamnolia vermicularis* were used as ingredients in incense powder mainly by the Sherpa (*N* = 30) and Lama (*N* = 11) ethnic groups, residing in Olangchung Gola, Ghunsa and Tsum valleys. They mixed these dried lichens with dried leaves of *Juniperus indica, J. squamata, Rhododendron anthopogon, R. decorum, R. lepidotum* and roots of *Jurinea dolomiaea.* They burn incense powder during their morning pray and religious ceremonies which releases pleasant and fragrant smoke.

*Usnea longissima*, an abundant, long thread-like lichen, was used for decoration by 17 respondents. This was being practiced among the Sherpa community of Olangchung Gola settlement of Kangchenjunga Conservation Area. The lichen was collected fresh and either framed making different shapes (designs) or simply hangs around in a room. Similarly, 16 respondents of Olangchung Gola, two hoteliers from Khumjung and one hotelier at Namche, Solukhumbu District were using fallen branches of trees with different species of lichens for the decoration in a corner of reception desks.

### Lichens and animal husbandry

One local healer and two respondents from Chekampar, Gorkha District, mentioned about the uses of lichens as an antiseptic and healing agents against external injuries in cattle. *Heterodermia diademata* is powdered and applied externally on fresh cuts and wounds. A local healer (Mr. Dorje Lundup Lama, age 68, personal communication) got this knowledge from his father and he believed this practice has been developed by observing practice of rubbing wounds on a stone by injured sheeps. Surprisingly, this information was not found in other research areas.

*Usnea longissima* is used as bedding materials for colt, chick, newly born goats and yak calves mainly by herders. Twenty-one respondents (male = 10, female = 11), from three Districts (Solukhumbu = 3, Gorkha = 6, Taplejung = 12), from three altitudinal levels (2600–3000 m = 2, 3000–3400 m = 17, ≥3400 = 2) collected about 200 kg (in average 9.5 kg per collector) of lichens in one season for this purpose. Interestingly, it was not used as bedding materials for human and rarely used as stuffing material for pillow, for reasons being unknown.

**Fig. 6:**
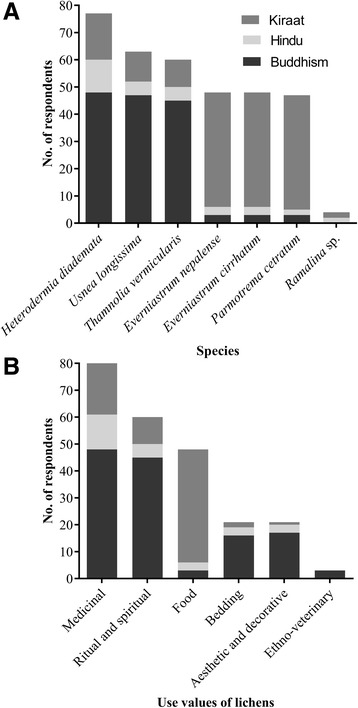
Respondents from three religious groups using **a** lichens species **b** for different use values

Among the six lichen uses which we identified in our research, food value (FV), medicinal value (MV), animal bedding value (BV) and ethno-veterinary value (EVV) include in provisioning services whereas ritual and spiritual values (RSV) and aesthetic and decorative values (ADV) relate with cultural services. From this study, it is found that the Kiraat religious group (Limbu and Rai) is using three lichen species (*Everniastrum nepalense, E. cirrhatum, Parmotrema cetratum*) mainly for their food value (FV) while the Buddhism religious group preferred lichens (*Heterodermia diademata, Usnea longissima,* and *Thamnolia vermicularis*) for medicinal (MV) and ritual and spiritual values (RSV). Only a low number of Hindu respondents (Brahman and Chhetri) were using lichens (Fig. 6).

## Discussion

Nepal is a multicultural country with 125 casts and ethnic groups and most of the caste/ethnic groups are spread throughout the 75 Districts of the Nepal [8]. They have their own ethnoecological knowledge and understanding regarding the surrounding environment and plant resources [30]. One plant may have several vernacular names. From this study, we have summarized 21 vernacular names which are used in different parts of Nepal. Among them, *Maangmaa*, *Myann* and *Lunhokya* from Dudhkunda valley, Sagarmatha are new additions. Lama et al. [15] reported seven vernacular names from Dolpa District, trans-Himalayan zone of Western Nepal in Himali Bhotia and Kham dialects (Table 2). Forest Regulations [31] has quoted lichens as *Budhani* and *Jhyauu*. So far, *Budhani* is not known as lichen by any ethnic names and in the literature published on lichens from Nepal [13, 32–44]. All the respondents in the studied areas mentioned *Jhyauu* for lichens and they have heard this name from their ancestors. So, here, we recommend using only a term *Jhyauu* rather than *Budhani* in the future. In an earlier publication by [15], *Thamnolia vermicularis* was mentioned as *Xylaria* sp. fungus from Dolpa, Nepal. This must be corrected in future studies. During our study, it was found that the vernacular name *Dankini Chyau* is the local name for lichens, but in contrary here *Chyau* means mushroom. So, it must be spelled as *Dankini Jhyauu.*

We have found that the Limbu ethnic group from Taplejung District is fond of preparing lichen soup, pickles, and sausages of *Everniastrum cirrhatum, E. nepalense,* and *Parmotrema cetratum.* The traditional knowledge how Limbu people prepare sausages with pig intestine, lichens and pig blood is unique. There are a few more lichen species around the globe which are used to prepare soup. Some major reports are *Cetraria islandica* [5], *Umbilicaria mammulata* [45], *U. muhlenbergii* [46], *Bryoria fremontii* [47]. *Cetraria islandica*, sold as “Iceland Moss” in the Scandinavian countries, is reported to be first lichen food for human beings, where lichen was mixed with grain flour to prepare bread, porridge, salads and several other local foods [5].

Traditionally, lichens are used to prepare indigenous foods, medicine, beverages, dyes, spices, decorations, animal feed and perfumes among different cultures across the world [4, 5, 14, 48–51]. In India, *Everniastrum cirrhatum* is used as holy material for sacrificial fire in ceremonies and also being used as spice and flavoring agent for vegetables and meats [52]. Similar use of lichens in India to prepare incense powder was also reported [53] without giving species and incense ingredients details.

*Lethariella cashmeriana, L. sernanderi, L. sinensis, Thamnolia vermicularis,* and *T. subuliformis* are used to prepare tea in the Yunnan Province of China [54], and *Cladonia rangiferina* in the Northern Hemisphere [55]. However, in the present study, we did not find such reports/publications from the field and published literature on Nepalese lichens. We have found substantial use of *Heterodermia diademata* in our research areas against cuts and wounds. This information is also matched by the information given by Saklani and Upreti [56] where they have mentioned about the uses of *H. diademata* in India among the Nepalese living in Gangtok (Sikkim) for cuts and to heal wounds. *H. diademata* together with *Eupatorium odoratum* for the treatment of fresh wound and cuts was reported among Limbu community of eastern Nepal [21]. In traditional herbal therapeutic practice in Far-western Nepal, lichen extract and decoction were applied to treat moles [17].

*Thamnolia vermicularis* is also used as antiseptic in Western Himalayas to kill buttermilk borne worms [57]. Uses of *T. vermicularis* among mountainous Sherpa community are more common than in the midhills communities. *Usnea* sp. are used as bedding materials and stuffing materials for the pillow and alternatively as mattresses at seasonal camps [58, 59], as we found in our sites.

There were no publications from Nepal reporting the uses of lichens with an ethno-veterinary purpose prior to this research, though there are few publications on uses of higher plants for the same purpose [60–63]. The use of *Usnea barbata* for the treatment of mammary infection in cattle is also reported from Xhosa, South Africa [64].

It was estimated that about 7,800 to 9,200 t lichens are collected to manufacture perfume in Morocco, Yugoslavia and France [65]. The uses of lichens to make dyes dates back to more than 3,500 years [66]. Many lichen species such as *Bryoria fremontii*, *Everniastrum cirrhatum, Letharia vulpina, Ochrolechia oregonensis, O. tartarea, Parmelia omphalodes*, *Parmotrema nilgherrensis, Rocella* spp., and others have been used as natural dyes from different parts of the World [5, 48, 67, 68]. We didn’t find such ethno-lichenological knowledge in our research area, although local women from Olangchung Gola, Ghunsa, Dudhkunda, Khumbu, Samagaun and Tsum valleys were active on weaving carpets using mixture of natural and synthetic dyes for the coloring of sheep wool.

Anthropogenic factors such as the unsustainable harvest and mismanagement in collection procedure of lichens at a large scale mainly to cover trade demands may reduce and threaten genetic resources. First of all, underlying mechanisms and processes of the lichens’ occurrence and dynamics should be understood so that suitable conservation strategies designed and recommendations for sustainable harvest can be made [69–71]. Epiphytic lichens play a crucial role in ecosystem functioning and structural complexity [72]. Environment Protection Act 1996 and Environment Protection Act Regulations 1997 have made Initial Environmental Examination (IEE) or Environmental Impact Assessment (EIA) mandatory in collection of selected plant species including lichen species in Appendix 1a, Article 8 [73]. A thorough analysis shows that IEA and EIA guidelines to reduce degradation of the environment are not followed by a larger group of people in the country due to lack of implementation and monitoring [74]. High-elevation lichens are also facing a significant threat due to climate change [75]. For epiphytic species with a high commercial value and demand such as *Everniastrum cirrhatum, E. nepalense* and *Parmotrema cetratum* future studies could test if in-situ culture techniques could support the production of substantial volumes of biomass for commercial harvest under climate change scenarios. Further, regular monitoring and certification system or ‘ecolabelling’ may improve sustainable harvesting systems [76].

## Conclusions

The present study reveals that lichens are of great interest as foods, traditional medicines or therapeutic values, aesthetic and spiritual values to the local inhabitants of different regions of Nepal. There is a long tradition of using lichens among the lichenophilic communities residing in the eastern mountainous parts of Nepal. Collection, consumption and marketing of lichens in sustainable ways would lead to long-term availability of these resources. Thus, it is recommended to carry out further research on abundance, stock estimation and collection impact on economically and socially important species.

## Additional file

Additional file 1: Table S1. Details of informants interviewed in three districts of Nepal. (DOCX 20 kb)

## Abbreviations

ADV: Aesthetic and decorative value
BV: Bedding value
CDB: Central Department of Botany
DNPWC: Department of National Parks and Wildlife Conservation
EIA: Environmental impact assessment
EVV: Ethno-veterinary value
FV: Food value
IEE: Initial environmental examination
KCAC: Kangchenjunga Conservation Area Committee
MV: Medicinal value
NTFPs: Non-timber forest products
RSV: Ritual and spiritual value
TLC: Thin layer chromatography
TUCH: Tribhuvan University Central Herbarium
VDCs: Village Development Committees
